# Association between Inflammation and New-Onset Atrial Fibrillation in Acute Coronary Syndromes

**DOI:** 10.3390/jcm13175088

**Published:** 2024-08-27

**Authors:** Ruxandra-Maria Băghină, Simina Crișan, Silvia Luca, Oana Pătru, Mihai-Andrei Lazăr, Cristina Văcărescu, Alina Gabriela Negru, Constantin-Tudor Luca, Dan Gaiță

**Affiliations:** 1 Cardiology Department, “Victor Babes” University of Medicine and Pharmacy, 2 Eftimie Murgu Sq., 300041 Timisoara, Romania; ruxandra.croicu@umft.ro (R.-M.B.); silvia.luca0@student.umft.ro (S.L.); oana.patru@umft.ro (O.P.); lazar.mihai@umft.ro (M.-A.L.); cristina.vacarescu@umft.ro (C.V.); constantin.luca@umft.ro (C.-T.L.); dgaita@cardiologie.ro (D.G.); 2 Institute of Cardiovascular Diseases Timisoara, 13A Gheorghe Adam Street, 300310 Timisoara, Romania; 3 Research Center of the Institute of Cardiovascular Diseases Timisoara, 13A Gheorghe Adam Street, 300310 Timisoara, Romania

**Keywords:** acute coronary syndrome, atrial fibrillation, inflammation

## Abstract

Acute coronary syndrome (ACS) is a complex clinical syndrome that encompasses acute myocardial infarction (AMI) and unstable angina (UA). Its underlying mechanism refers to coronary plaque disruption, with consequent platelet aggregation and thrombosis. Inflammation plays an important role in the progression of atherosclerosis by mediating the removal of necrotic tissue following myocardial infarction and shaping the repair processes that are essential for the recovery process after ACS. As a chronic inflammatory disorder, atherosclerosis is characterized by dysfunctional immune inflammation involving interactions between immune (macrophages, T lymphocytes, and monocytes) and vascular cells (endothelial cells and smooth muscle cells). New-onset atrial fibrillation (NOAF) is one of the most common arrhythmic complications in the setting of acute coronary syndromes, especially in the early stages, when the myocardial inflammatory reaction is at its maximum. The main changes in the atrial substrate are due to atrial ischemia and acute infarcts that can be attributed to neurohormonal factors. The high incidence of atrial fibrillation (AF) post-myocardial infarction may be secondary to inflammation. Inflammatory response and immune system cells have been involved in the initiation and development of atrial fibrillation. Several inflammatory indexes, such as C-reactive protein and interleukins, have been demonstrated to be predictive of prognosis in patients with ACS. The cell signaling activation patterns associated with fibrosis, apoptosis, and hypertrophy are forms of cardiac remodeling that occur at the atrial level, predisposing to AF. According to a recent study, the presence of fibrosis and lymphomononuclear infiltration in the atrial tissue was associated with a prior history of AF. However, inflammation may contribute to both the occurrence/maintenance of AF and its thromboembolic complications.

## 1. Introduction

Acute coronary syndrome (ACS) is a complex syndrome that refers to a spectrum of clinical entities, including unstable angina, ST segment elevation myocardial infarction (STEMI), and non-ST segment elevation myocardial infarction (NSTEMI), representing a significant public health burden worldwide, with a significant impact on morbidity and mortality rates [1]. The major pathophysiologic mechanism underlying the development of ACS involves coronary plaque disruption, with successive platelet aggregation and thrombosis. In atherosclerotic lesions, vascular inflammation involves both proatherogenic and antiatherogenic immune networks, which contribute to plaque destabilization and the progression of acute coronary events [2]. In the pathogenesis of atherosclerosis, endothelial dysfunction plays a critical role by facilitating the uptake of low-density lipoprotein (LDL), particularly in plaques that are prone to disruption [3,4,5]. The endothelial inflammatory response amplifies the activation of both innate and adaptative immunity. Moreover, inflammatory signaling provides the release of cytokines with a significant role in the process of plaque rupture [6]. Various inflammatory biomarkers, such as C-reactive protein and interleukins, have been demonstrated to be predictors of prognosis in patients with ACS. The majority of patients with ACS have a high level of high-sensitivity C-reactive protein, a biomarker of systemic inflammation and a predictive factor for high cardiovascular mortality [7]. The inflammatory response, through cell signaling activation and atrial fibrosis, is thought to be involved in the appearance of new-onset atrial fibrillation (NOAF), as a common arrhythmic complication in the setting of acute coronary syndromes [8]. Atrial remodeling, as a result of inflammatory status, acts as a substrate for AF initiation and maintenance [9]. The multitude of changes in the atrial structure, including atrial dilatation, atrial cardiomyocyte hypertrophy, and fibrosis, can lead to action potential shortening, reduced electrical connections between cells, and alteration in Ca^2+^ handling [10]. Transthoracic echocardiography (TTE) plays an important role in the assessment of the cardiac structure and function, guiding the therapeutic management of patients with ACS who develop NOAF. Several studies have revealed that the left atrial diameter, right atrial diameter, and heart failure are independent predictive risk factors of NOAF in patients with acute coronary syndrome [11]. In STEMI patients, the left ventricular ejection fraction (LVEF) was significantly reduced and an elevated left ventricular filling pressure was found. Prospective studies showed that left atrial strain (LAS) parameters were associated with the occurrence of NOAF, revealing that left atrial dysfunction is a result of the increased left ventricular filling pressures and volume overload due to a decreased LVEF following STEMI [12]. The etiology of NOAF is multifactorial, including abnormalities in the autonomic nervous system, due to an imbalance between an increased sympathetic tone and decreased vagal activity, localized and systemic inflammation, and hormonal activation. However, many studies have concluded that NOAF during hospitalization significantly increases the risk of major adverse cardiac events and mortality [11]. Although in most cases, atrial fibrillation during ACS management is tolerated, the current European Society of Cardiology (ESC) Guidelines for the management of acute coronary syndromes recommend prompt treatment by using antiarrhythmic therapy and electrical cardioversion for patients with acute hemodynamic instability. According to observational studies, patients with ACS and AF are less likely to receive the appropriate antithrombotic therapy and more likely to develop major complications than patients without AF [13]. The ESC Guidelines for the diagnosis and management of atrial fibrillation recommends the use of antithrombotic therapy, including oral anticoagulation, preferably non-vitamin K antagonist oral anticoagulant (NOAC), in association with a P2Y12 inhibitor, due to the significantly less major bleeding occurring than with triple therapy. However, current evidence reveals that a short course of triple therapy could be beneficial for some AF patients at an increased risk of ischemic events after a recent ACS or those undergoing PCI. Due to a greater risk of major bleeding, the use of prasugrel or ticagrelor should be avoided in ACS patients with AF [14].

## 2. Inflammation in the Pathogenesis of ACS

### 2.1. Adaptive Immunity

As a chronic inflammatory disorder, atherosclerosis is characterized by a dysfunctional immune response, involving interactions between immune (macrophages and T lymphocytes) and vascular cells (endothelial cells and smooth muscle cells) [15,16,17]. In approximately 75% ACS cases, coronary thrombosis is driven by plaque rupture. The inception of plaque rupture occurs within a vulnerable plaque, characterized by a thin fibrous cap overlaying a lipid-laden core [11,18]. Mechanical stress and degradation of the extracellular matrix are among the factors precipitating an acute event. A prolonged inflammatory status contributes to the release of proteolytic enzymes, particularly matrix metalloproteinases (MMPs), which degrade components of the extracellular matrix and weaken the fibrous cap, increasing the susceptibility to plaque rupture [19]. A recent histological analysis of atherosclerotic coronary arteries showed that unstable atherosclerotic plaques are characterized by the presence of macrophages, lymphocytes, and mast cells [20]. The dominant cell types at the site of plaque erosion are macrophages and T lymphocytes. When activated, these inflammatory cells promote inflammation at the site of plaque disruption by releasing cytokines that have the potential to activate the endothelium, transforming its natural antiadhesive and anticoagulant properties. According to many studies, the architecture and cellular composition of the underlying atherosclerotic plaques were involved in complicated coronary artery lesions causing acute myocardial infarction, suggesting that an active inflammatory process contributes to plaque rupture or erosion [21,22]. Inflammatory cytokines contribute to improved smooth muscle cell reactivity to local vasoconstrictors by reducing matrix synthesis and increasing the degradation and synthesis of endothelin. These vulnerable parts of plaques are areas of predilection for inflammation due to the high number of activated mast cells. Mast cells represent a cell type related to the inflammatory process that induces matrix degeneration by the formation of tryptase and chymase [20,23]. The involvement of the inflammatory process in the progression of acute coronary events is suggested by the presence of activated mast cells in the layer of infarct-related coronary vessels and by the high number of mast cell densities in normal segments of the infarct-related artery [24,25]. Activated platelets release inflammatory mediators that support the chemotaxis, adhesion, and migration of leukocytes to areas of inflammation [26,27,28]. Platelet activation contributes to the recruitment of leukocytes and the development of circulating platelet–leukocyte aggregates, the protagonists of inflammatory reactions in the vessel wall [29]. Platelets play an important role in thrombus formation and atherosclerotic inflammation by modulating monocyte migration and differentiation to macrophages [30,31,32]. M1-type macrophages are pro-inflammatory cells stimulated by monocyte-colony-stimulating factor (M-CSF) and mediated by platelets to express high levels of inflammatory cytokines (interleukins IL-6, IL-1β, and IL-18) and an increased production of reactive oxygen species [33,34]. M2-type macrophages contribute to tissue remodeling, a ngiogenesis, and immune regulation, inducing the differentiation of regulatory T cells that secrete anti-inflammatory cytokine transforming growth factor-β (TFG-β) [34,35]. By the expression of the Forkhead box P3 transcription factor (FOXP3), regulatory CD4(+)T cells prevent excessive immune responses. In addition, a randomized cohort study suggested that low levels of CD4(+) FOXP3 T regulatory cells are associated with an increased risk for the development of myocardial infarction [36,37,38]. Studies on M1 and M2 macrophages have suggested that M1 macrophages promote plaque inflammation, while M2 macrophages limit it. T lymphocytes, especially TH1 cells, have inflammatory functions by stimulating interferon-*γ* (IFN-*γ*) production and, consequently, affecting the production of interstitial collagens in vascular smooth muscle cells (VSMCs), providing the thin fibrous cap susceptible to ACS [36,39]. A unique subset of T lymphocytes, CD4(+) CD28(null) T cells, have the ability to release inflammatory cytokines and cytotoxic molecules, amplifying inflammatory pathway increases in patients with acute coronary syndrome [40,41]. A recent study concluded that high levels of CD4(+) CD28(null) T cells are associated with poor prognoses in patients with an inflammatory status [42,43]. This contribution to apoptosis resistance may be due to the imbalance of proapoptotic molecules (Bim, Bax, and Fas) and antiapoptotic molecules (BcI-2 and BcI-xL) demonstrated by Kovalcsik in a recent study and the fact that their immune response cannot be suppressed by regulatory T cells. Furthermore, Hammer et al. hypothesized a possible mechanism of CD4(+) CD28(null) T cells on the development of atrial fibrillation, underlying the implication of the inflammatory process in the disease progression [34].

### 2.2. Innate Immunity

As modulators of macrophage progression, Netrin-1 and semaphorin-3 inhibit the migration of macrophages by binding to CC motif chemokines (CCL19 and CCL21) [44]. The main action of netrin-1 on macrophage modulation has been demonstrated in animal studies, suggesting that the intentional lowering of Netrin-1 concentrations can lead to increasing macrophage efflux [45]. Furthermore, the first human study on Netrin-1 and ACS revealed that Netrin-1 levels increased in the early phase of ACS and decreased in patients for whom reperfusion was established after angiography, demonstrating the importance of Netrin-1 as a biomarker indicator of diagnosis and successful reperfusion in ACS [46]. Different classes of cytokines, including the tumor necrosis factor-alpha (TNF-α) family, interleukin-1 family, chemokines, and interferons, are involved in the process of the destabilization of atherosclerotic plaques and, consequently, in the progression to ACS [47,48]. An important component of the innate immune system is the nucleotide-binding leucine-rich repeat-containing pyrin receptor 3 (NLRP3) [49,50]. In ACS, NLRP3 triggers the immune cell to release inflammatory cytokines, IL-1β and IL-18, which destabilize the plaque by upregulating vascular cell adhesion molecules (VCAMs) and promoting pro-inflammatory reactions [51]. Interleukin-6 (IL-6) plays an important role in innate immunity, assuming physiological functions associated with immune cell regulation, proliferation, and differentiation [52,53]. Among the acute-phase reactants released by the liver in response to IL-6, fibrinogen participates directly in thrombus formation. Studies have shown that the serum fibrinogen-to-albumin ratio (FAR), a biomarker for inflammation and thrombosis, has been used to predict the severity and prognosis of NOAF during hospitalization in patients with AMI after percutaneous coronary intervention (PCI) [54].

## 3. Atrial Remodeling

New-onset atrial fibrillation is the most prevalent and common arrhythmia in the setting of ACS, with a reported incidence ranging from 2.4% to 37%. The increased occurrence of atrial fibrillation in ACS can be explained by several mechanisms, such as ischemia, a reduction in the atrial blood flow, and higher left ventricle end-diastolic and left atrial pressures [11]. Numerous evidence shows a close relationship between inflammation and atrial fibrillation, suggesting that the initiation of AF is a consequence of the necrosis and fibrosis caused by inflammatory processes, which can trigger atrial dysrhythmias directly through fluctuations in membrane potential [10,55]. Elevated levels of pro-inflammatory cytokines are associated with the development of AF in the general population, and it is well-known that more pronounced inflammatory activity represents a strong risk factor for thromboembolic events [56]. A chronic pro-inflammatory status is a general feature of aging, and according to studies, the risk of developing AF doubles with each progressive decade and exceeds 20% by the age of 80 years [57]. In ACS patients, inflammation has been suggested as a pathophysiological mechanism in the development and perpetuation of AF. Chronic inflammation in elderly people, usually known as inflammaging, accelerates the development of structural atria damage, leading to fibrosis and a high risk of AF occurrence. A recent retrospective observational cohort study demonstrated that age is an independent predictor factor for the development of NOAF in ACS patients [58]. Moreover, in these patients, frailty plays an important role, being a major predictor of adverse outcomes in older patients with coronary artery disease, and should always be assessed [59]. The results of atrial biopsies taken from patients with AF compared with controls have demonstrated evidence of inflammatory infiltrates within the atrial tissue [60]. During ischemia, apoptotic cells are replaced by collagen infiltrates, a process stimulated by cardiac fibroblasts and defined as reparative fibrosis. In response to inflammation, cardiac fibroblasts are activated by inflammatory mediators and growth factors, causing fibrosis formation in the atrial tissue [61]. This remodeling includes both structural and functional changes in the atrial myocytes in response to stimuli.

### 3.1. Structural Remodeling

**Figure 1:**
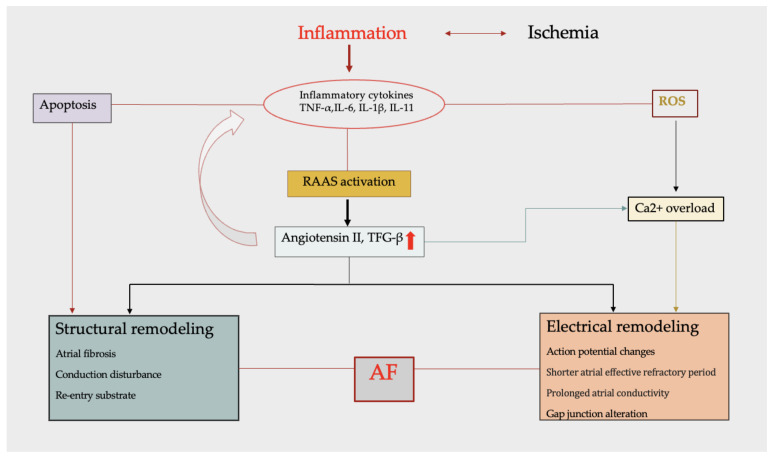
Major components of atrial remodeling. The inflammatory process promotes the production of pro-inflammatory cytokines (IL-6, IL-8, TNF-α, and IFN-γ) contributing to both structural and electrical remodeling. Inflammatory cytokines induce structural remodeling by activation of cardiac fibroblasts, leading to apoptosis and atrial fibrosis. Stimulated by inflammatory status, RAAS increases the production of Angiotensin II and TGF-β, which assess the secretion of more cytokines, maintaining inflammation. Among ROS, Angiotensin II contributes to abnormal Ca^2+^ handling, resulting in a decreased action potential duration and electrical remodeling. Abbreviations: AF—atrial fibrillation; ROS—reactive oxygen species.

AF develops in the context of an altered myocardial environment due to myocytes and interstitial changes. Structural changes in the atrial myocardium include left atrial enlargement and increasing atrial fibrosis. Thus, by promoting conduction disturbances, atrial fibrosis is a central component of atrial remodeling in atrial fibrillation [62,63]. This altered impulse conduction is characterized by a slower depolarization wave with an electrical impulse that propagates through an alternative pathway, leading to multiple reentrant circuits [64]. Inflammation is one of the multiple factors that can lead to cardiac fibrosis. The inflammatory process promotes the production of pro-inflammatory cytokines, playing an important role in the pathogenesis of atrial fibrosis. The expression and activation of inflammatory cytokines by myeloperoxidase (MPO) and heat-shock proteins (HSPs) induce atrial fibrosis and apoptosis, leading to slowed conduction [65]. The renin–angiotensin–aldosterone system (RAAS) is involved in the pathogenesis of atrial fibrillation, initiating structural and electrical remodeling (Figure 1). The RAAS contributes to atrial fibrosis and structural remodeling by expressing mitogen-activated protein kinase (MAPK) and increasing the production of TGF-β and the extracellular matrix protein [66].

Different studies have demonstrated the pro-inflammatory role of Angiotensin II by inducing neutrophil recruitment and the production of cytokines such as, IL-6, IL-8, TNF-α, and IFN-γ, with a significant implication in the management of ACS and AF. Increased levels of Angiotensin II and activated TGF-β induce the expression of profibrotic molecules in cardiac fibroblasts, promoting collagen synthesis and, eventually, atrial fibrosis and atrial enlargement [67]. Recently, some studies revealed that the left atrial diameter is considered to be an independent predictor factor for NOAF during ACS [11].

### 3.2. Electrical Remodeling

The electrical remodeling of the atria occurs in parallel with the changes occurring in structural remodeling. Many theories assess the alterations in multiple re-entrant circuits originating in the atria, conducted at various velocities through tissues with different refractory periods. These types of remodeling can lead to triggered activity and electrical re-entry, which are major mechanisms of AF initiation [68]. Electrical remodeling creates a re-entry substrate, leading to premature impulses. The main components of electrical remodeling include action potential changes, with shorter atrial effective refractory periods and prolonged atrial conductivity, impaired Ca^2+^ handling, and gap junction remodeling [69]. As a part of the RAAS system, Angiotensin II and reactive oxygen species (ROSs) contribute to abnormal Ca^2+^ handling and, thus, to remodeling promotion. The Ca^2+^ overload shortens the atrial effective refractory period and demands re-entry circuits. Ca^2+^-induced activation suppresses the gene expression of L-type Ca^2+^ channel subunits, resulting in a decreased action potential duration and electrical remodeling [63,70,71].

## 4. Inflammatory Biomarkers

### 4.1. Interleukin-1 (IL-1)

Interleukin-1 is a pro-inflammatory cytokine that comprises two major isoforms, IL-1α and IL-1β. In particular, IL-1β is involved in the pathogenesis of coronary atherosclerotic diseases by initiating several processes leading to the formation, growth, and rupture of atherosclerotic plaques. In hypoxic cells, the NLRP3 inflammasome activates IL-1β, which modulates the lipid metabolism and enhances atherogenesis by promoting vascular smooth cell proliferation [72,73,74]. The Canakinumab Anti-inflammatory Thrombosis Outcome Study (CANTOS) revealed the potential implication of IL-1β in conditioning the clinical course of patients after an acute coronary syndrome, concluding that the IL-1β innate immunity pathway with canakinumab significantly reduced the recurrence of new cardiovascular events [75,76]. Another study on post-MI patients demonstrated that IL-1β signaling in the bone marrow indirectly affects leukocyte production. By neutralizing IL-1β, the blood levels of inflammatory monocytes and neutrophils started to decrease, reducing leukocytosis and contributing to the resolution of inflammation [77]. The activation of the IL-1β pathway, different from the NLRP3 inflammasome, could help to assess the appearance of NOAF in patients with ACS. Recent studies demonstrated that pressure overload increased the gene expression of both IL-1β and MCP-1, leading to sustained AF [78,79].

### 4.2. Interleukin-6 (IL-6)

IL-6 is a pro-inflammatory cytokine, primarily produced by macrophages and T cells, with notable prognostic value in patients with ACS [80]. Cardiac fibroblasts release IL-6 and IL-1 as a response to oxygen deprivation in the ischemic region. Interleukin-1 and tumor necrosis factor (TNF) can induce IL-6 expression, which stimulates hematopoiesis, creating a consistent contribution to the leukocytosis that can accompany ACS [81]. IL-6 improves local and systemic inflammation by activating leukocytes and signaling hepatocytes to produce acute-phase reactants. In ACS, IL-6 promotes thrombus formation and stability by initiating the production of fibrinogen, a precursor of thrombi, and plasminogen activator 1 (PAI-1), an inhibitor of fibrinolysis. IL-6 contributes to atrial remodeling and induces a pro-thrombotic state by increasing platelet production and coagulation factors, predisposing to AF (Table 1) [82,83]. Cohort studies showed that elevated IL-6 levels were significantly associated with a higher risk of stroke and all-cause mortality in patients with AF [84]. Furthermore, serum IL-6 levels were independently related to adverse events and mortality during long-term follow-up (up to 2 years) in a large cohort of anticoagulated permanent/paroxysmal AF patients [85].

### 4.3. Interleukin-8 (IL-8)

Interleukin- 8 is a neutrophil-specific chemotactic factor that plays an important role in the inflammatory response that contributes to the development of atherosclerotic plaques [86]. Expressed in macrophage-rich areas of atherosclerotic lesions, IL-8 has proatherogenic effects by triggering the adhesion of monocytes to the vascular endothelium, modulating the platelet–platelet and platelet–leukocyte interactions associated with thrombogenesis [87]. Studies showed that the IL-8 level was a powerful predictor of cardiac events and restenosis in patients with ACS who required percutaneous coronary intervention (PCI). Dominguez-Rodriguez et al. concluded that increased serum levels of IL-8 after PCI are probably a predictor for the development of heart failure in patients with acute myocardial infarction, suggesting the involvement of inflammation in the prognosis of these patients [88]. Case–control studies demonstrated that the interleukin-8 levels are elevated in the sera of patients with AF [89]. Another cohort-type study of 113 patients undergoing CABG surgery revealed that higher concentrations of IL-8 in CABG patients with postoperative AF are associated with an inflammatory status in the pathogenesis of postoperative AF after open-heart surgery [90]. This analysis suggests a possible link between inflammation and NOAF, but further investigation is still needed.

### 4.4. Interleukin-11 (IL-11)

Interlukin-11 is a member of the IL-6 family cytokines involved in the pathogeneses of inflammatory diseases. Studies have shown that IL-11 promotes fibrosis of the heart, lungs, and liver. In addition, experimental analysis revealed that the inhibition of IL-11 reduced vascular fibrosis in mice. In inflammatory conditions, TGF-β and IL-1 interfere with the expression of IL-11, increasing the risk of developing restenosis after stent implantation [91]. IL-11 has a specific effect on hematopoietic cells, being responsible for platelet production; therefore, recombinant human IL-11 (rhIL-11) has been used as an anti-inflammatory agent to treat chemotherapy-associated thrombocytopenia. A recent study concluded that rhIL-11 therapy increases the occurrence of atrial fibrillation in these patients by shortening the atrial refractory period and inducing atrial remodeling [92].

### 4.5. Interleukin-17A (IL-17A)

In the pathogenesis of ACS, IL-17A promotes ADP-induced platelet activation and aggregation through the mitogen-activated protein kinase (MAPK/Erk2) signaling pathway. Zhang et al. concluded that the concentration of serum IL-17A and the platelet aggregation levels were obviously elevated in ACS patients compared with stable angina (SA) patients [93]. Moreover, studies have shown that IL-17A might stimulate the release of pro-inflammatory cytokines, including IL-1β, TGF-β, and IL-6, which also participate in the pathogenesis of myocardial fibrosis and promote the occurrence of AF [94,95].

### 4.6. C Reactive Protein (CRP)

**Figure 2:**
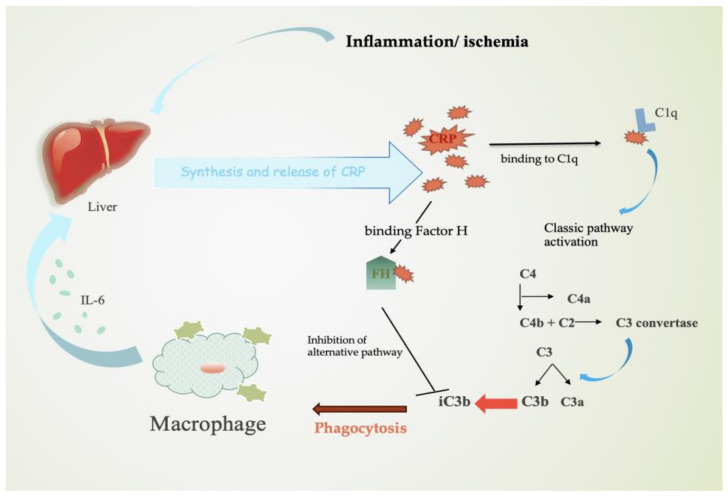
Mechanism of CRP in inflammation. Stimulated by IL-6, hepatocytes release CRP molecules and activate the classic complement pathway. By binding Factor H, the alternative pathway is inhibited and, therefore, the inactivation of C3b to iC3b promotes phagocytosis. Abbreviations: CRP—C reactive protein.

C reactive protein is an inflammatory marker that has been linked to the pathogenesis and prognosis in patients with coronary artery disease, congestive heart failure, and AF. In ACS, CRP is synthesized by hepatocytes in response to pro-inflammatory cytokines, particularly IL-6. CRP activates the classic complement pathway by binding to lyso-phosphatidylcholine (LPC) and consecutively to C1q. By binding Factor H, the alternative complement pathway is inhibited and, therefore, the inactivation of C3b to iC3b occurs (Figure 2). In healthy cells, complement activation does not take place, due to C3b’s rapid inactivation to iCb3. In ischemic/inflamed cells, due to damaged surfaces, the process of CRP–Factor H binding is decreased. The activation of the classic complement pathway mediated by CRP results in a higher number of iCb3 molecules that promote phagocytosis [96].

Many studies have demonstrated that CRP might affect coronary artery disease progression by activating the complement pathways and platelets and suppressing fibrinolysis. Further analysis concluded that high-sensitivity CRP (hs-CRP) levels are directly proportional to cardiovascular risk and the magnitude of the inflammatory response to myocardial ischemia [97]. However, the correlation between inflammation and atrial fibrillation is supported by different studies that have related inflammatory biomarkers to arrhythmia. The circulating levels of CRP are increased in patients with AF, suggesting a possible involvement of the inflammatory status in the occurrence of NOAF. Bruins et al. were the first to propose the inflammation–AF correlation, revealing that the peak incidence of AF coincided with the peak elevation of CRP levels in patients with coronary artery bypass surgery [98]. Four-year follow-up data explored the CRP-related incidence of AF, concluding that patients with high CRP and complement levels had a significantly higher risk of AF than patients with normal CRP and low complement levels. This study revealed a possible implication of the complement activation pathway and CRP mechanism in the development of NOAF in ACS patients. Moreover, recent data show that CRP significantly increased the Ca^2+^ in atrial myocytes without affecting the expressions of gene-encoding pro-collagens in atrial fibroblasts, suggesting a different mechanism of CRP in the development of AF [55].

### 4.7. Tumor Necrosis Factor Alpha (TNF-α)

TNF-α is a pro-inflammatory cytokine involved in the pathogenesis of ACS, contributing to plaque destabilization and progression. Induced by T cells, TNF-α increases the production of other inflammatory cytokines and initiates the down-regulation of cardiac proteins affecting the myocardial function. Increasing evidence suggests that inflammatory biomarkers, including TNF-α, are elevated in patients with AF [99]. Studies on animals have revealed the fibrotic action of TNF-α in the development of atrial fibrosis by mediating the TGF-β/Smad signaling pathway and the activation of cardiac fibroblasts [100]. The conversion of cardiac fibroblasts into myofibroblasts generates the production of MMPs, resulting in extracellular matrix degradation and an altered atrial structure. In addition, both hs-CRP and TNF-a have been shown to upregulate angiotensin type 1 receptors in vascular and cardiac fibroblasts, underlying the correlation between inflammation and atrial fibrillation [101].

### 4.8. Galectin-3 (Gal-3)

Galectin-3 is a soluble B-galactoside-binding lectin that mediates the inflammatory and fibrosis pathways. Galectin-3 is expressed by macrophages and contributes to fibrosis by inducing fibroblast proliferation and collagen synthesis. According to recent studies concerning the pathogenesis of ACS, Galectin-3 has been proven to have an influence on plaque formation and destabilization. Even if the exact role of Gal-3 in the process of ACS is unknown, elevated levels were observed during the acute phase of AMI [102]. Many studies have revealed that Gal-3 is a main component of ventricle remodeling and early prognosis after AMI. Soltan et al. concluded that there is a strong correlation between Gal-3 levels and the severity of coronary artery disease in patients with ACS, highlighting its potential as a promising biomarker for the diagnosis of coronary artery disease [103]. A recent analysis of patients with AMI revealed that elevated levels of galectin-3 were associated with a 4.4 times higher risk of developing atrial fibrillation, suggesting that Gal-3 could be a prognostic biomarker in acute myocardial infarction. The same study showed that patients with AF had higher levels of CRP (*p* < 0.01) and Gal-3 (*p* < 0.05) than those without AF, highlighting the implication of inflammatory status in the development of this pathology [104]. Furthermore, it has been shown that Gal-3 is involved in the atrial remodeling process by inducing cardiac fibroblasts to proliferate and deposit type I collagen in the myocardium [105].

### 4.9. Thrombospondin-1 (TSP-1)

Thrombospondin-1 is a multi-modular glycoprotein, mainly produced by macrophages and platelets, with an important role in the inflammatory response and fibrogenesis. By binding the CD47 receptor, TSP-1 stimulates ROS production in VSMCs and promotes vascular dysfunction, contributing to atherosclerotic lesions [106]. Experimental data have demonstrated that TSP-1 is expressed along the border of infarcted areas, which can be increased by ischemia/reperfusion [107]. Studies have shown that ACS patients with decreased TSP-1 levels have a low probability of acute events [108]. Liao et al. analyzed 219 patients with AMI and no previous arrhythmias who underwent percutaneous coronary intervention, which may serve as a potential indicator of atrial arrhythmias post AMI [109]. Recent data suggest that TSP-1 is involved in atrial remodeling and fibrosis through the TGF-β1 pathway [110], but its exact mechanism in the pathogenesis of AF is not known.

### 4.10. B-Type Natriuretic Peptide (BNP)

BNP is a natriuretic hormone with an important role in vasodilation [111]. Experimental studies have demonstrated a correlation between BNP and inflammation, suggesting that pro-inflammatory cytokines, such as IL-1β and TNF-α, target the BNP gene and increase BNP mRNA in cultured cardiomyocytes [112]. Used mainly for the diagnosis of heart failure [113,114], BNP levels contribute significantly to the diagnostic efficiency of NSTEMI among necrosis markers upon admission, according to three major studies. Even if the mechanism underlying this is not completely elucidated, it was suggested that, in response to myocardial injury and increased ventricular wall stress, cardiomyocytes could release BNP directly. This was demonstrated by the elevated levels of BNP observed soon after cardiac ischemia, underlying its implication in the pathogenesis of ACS [115]. A retrospective cohort study on patients with ACS concluded that BNP levels were an independent predictor for NOAF during hospitalization [11]. Furthermore, Asanin et al. established that BNP independently predicts the occurrence of NOAF in STEMI patients treated by primary PCI [116].

## 5. Echocardiographic Assessment and Predictive Risk Factors for NOAF

Transthoracic echocardiography (TTE) plays an important role in the assessment of patients with ACS who develop NOAF, providing comprehensive information on the cardiac structure and function. TTE evaluates the systolic and diastolic left ventricular function, left ventricular volume, and left and right atrial diameters. Several studies have revealed that the left atrial diameter, right atrial diameter, and heart failure are independent predictive risk factors for NOAF in patients with acute coronary syndrome [9]. De Vos et al. suggested that, in order to prevent the development of and complications in AF, tissue Doppler imaging, PA-TDI, which estimates the time from the initiation of the P wave on the ECG (lead II) to the A′ wave on the lateral left atrial tissue, may be used to identify patients at risk. Moreover, the same study concluded that a prolonged PA-TDI interval may predict the development of NOAF [117].

A prospective echocardiographic study on patients with STEMI concluded that left atrial strain (LAS) during the reservoir phase (LASr) in systole is an independent predictive factor for NOAF. In these patients, the left ventricular ejection fraction (LVEF) was significantly reduced and an elevated left ventricular filling pressure was found. In the study, all LAS parameters were associated with the occurrence of NOAF, revealing that among the factors contributing to left atrial dysfunction are the increased left ventricular filling pressures and volume overload resulting from the decreased LVEF following STEMI. This increased pressure and volume can lead to left atrial dilation and stretching, reducing the LASr function. In addition to volume overload, ischemia and inflammation in the left atrial myocardium can contribute to the development of left atrial tissue fibrosis and remodeling [12].

## 6. Gaps in Evidence and Future Perspectives

Many studies have suggested the involvement of the inflammatory process in the occurrence of NOAF in ACS patients, but a specific biomarker has not been established. Although the levels of hs-CRP may correspond to the risk of developing NOAF, it should be known that increased levels can coexist in patients with ACS [97]. Furthermore, BNP levels independently predict NOAF in patients with ACS, emphasizing the fact that inflammation is not involved in this process [115].

A specific therapeutic algorithm cannot yet be proposed outside the actual guidelines’ recommendations. Current anti-inflammatory therapies have tried to approach the most important mediators of inflammation specifically, IL-1β and IL-6 chemokines, IFN-γ, and TNF-α. Large randomized clinical studies and meta-analyses have shown that anti-inflammatory therapies have a favorable efficacy profile and can reduce the hazard of cardiovascular events [118].

The CANTOS study showed that isolated therapies targeting inflammation (IL-1β inhibition) can reduce the risk of cardiovascular events and may be useful in patients post-ACS. However, the increased risk of non-cardiac complications and the non-significant effect on all-cause mortality have increased the need for the development of other novel therapeutic strategies, as well as for an improved selection of patients more likely to respond to anti-inflammatory therapies [76]. Therapies that express the effect of TNF-α inhibitors in atherosclerosis, such as infliximab, etanercept, and adalimumab, have been evaluated in studies and have shown anti-inflammatory properties. The mechanism is hypothesized to be significant in promoting atherosclerosis, but data regarding the effect of TNF-α inhibitors on the cardiovascular risk of patients with ACS are insufficient. Anakinra, a recombinant IL-1 receptor antagonist used as a treatment in several inflammatory diseases, could potentially reduce vascular inflammation in patients with coronary artery disease; however, according to two randomized studies, anakinra did not reduce the risk of cardiovascular events. The NLRP3 inflammasome is significant for the production of pro-inflammatory cytokines and, as such, its inhibition could be a therapeutic target. In AMI mice studies, MCC950, a potent selective inhibitor of the NLRP3 inflammasome, reduced myocardial fibrosis, but further examinations need to be conducted. Currently, a large randomized trial proposing the examination of the therapeutic role of IL-6 inhibition is ongoing, and its potential could be promising [118].

Multiple recent studies have evaluated the involvement of the inflammatory process in the prognoses of acute coronary syndromes, suggesting that a small dose of colchicine can provide a significant decrease in the mortality risk of cardiovascular causes, as well as the revascularization rate subsequent to myocardial infarction. Thus, the ESC Guidelines for the management of acute coronary syndromes from 2023 recommend the administration of a small dose of colchicine, from recommendation class IIB level of evidence A, in patients diagnosed with acute coronary syndromes, in which the associated risk factors cannot be optimally controlled or in the case of the recurrence of major cardiovascular events. Moreover, retrospective studies revealed that Angiotensin II receptor blockers could prevent NOAF in patients with left ventricular dysfunction or hypertension. Evidence from randomized controlled trials revealed that mineralocorticoid receptor antagonist (MRA) therapy can reduce new-onset atrial arrhythmias in patients with heart failure with reduced ejection fraction (HFrEF), but further investigations regarding precise action on NOAF should be performed [13].

Beta-blockers, due to their antiarrhythmic properties, represent a class of drugs that could impact the incidence of NOAF in patients with ACS. The widespread use of beta blockers post-AMI is based mostly on clinical studies published in the pre-reperfusion era. However, recent data suggest that the benefits and the optimal duration of beta-blockers in patients without a reduced EF or heart failure after an ACS are questionable [64,119,120]. The actual impact of reducing the use of beta-blockers in patients with a preserved EF and AMI is difficult to quantify, and there is still a need for both randomized trials and real-world registries to clarify this aspect. Moreover, further research should better stratify patients at risk of NOAF in order to expand the potential use of beta-blockers beyond the echographic assessment of the ejection fraction.

## 7. Conclusions and Main Key Findings

Acute coronary syndrome is a major cause of death worldwide. New growing evidence has implicated the involvement of the inflammatory process in the development and prognosis of ACS due to its major contribution to the thrombus formation process. Both innate and adaptative systems play critical roles in atherosclerosis and thrombotic complications by mediating the interactions between immune and vascular cells. Possible risk factors have been studied and associated with NOAF in ACS patients (Table 2).

Many studies have highlighted that fibrosis, inflammation, and myocardial dysfunction are closely related and involved in the occurrence of NOAF in AMI. Through different pathways, inflammatory cytokines released in response to inflammation are involved in both the structural and electrical remodeling of the atrial tissue. These disturbances in atrial conduction could be the main factors contributing to the occurrence of NOAF in patients with ACS, but the certain pathophysiological mechanism underlying the influence of inflammation on the occurrence of atrial fibrillation has not yet been elucidated. One important issue remaining is the identification of inflammatory biomarkers that could predict the appearance of NOAF and, thus, many inflammatory biomarkers have been evaluated to play major roles in the initiation of NOAF during hospitalization. However, current knowledge suggests that inflammation may facilitate the occurrence of NOAF. Also, BNP independently predicts the occurrence of NOAF in STEMI patients treated by primary PCI, emphasizing that the acute LA pressure and volume overload caused by ACS may induce NOAF, without the involvement of inflammation. Thus, for a better understanding of the association between inflammation and NOAF in ACS patients, further studies are needed in order to assess the predictive value of inflammatory biomarkers, especially CRP and interleukins.

## Figures and Tables

**Table 1:** Role of interleukins in the pathogenesis of atrial fibrillation.

Interleukins	Role in Pathogenesis of AF
Interleukin-1 Interleukin-6 Interleukin-11	Induces atrial remodeling Pressure-overload-induced sustained AF Induces atrial remodeling Atrial myocardium inflammatory status Shortening of atrial refractory period
Interleukin 17A	Stimulates cardiac fibroblast proliferation and promotes the occurrence of AF

**Table 2:** Main key findings.

Age, left atrial diameter, right atrial diameter, and heart failure are independent risk factors of developing NOAF in ACS patients
LASr is an independent predictive factor of NOAF in STEMI patients
Patients with high levels of CRP, TNF-α, and complement levels had a significantly higher risk of AF
IL-6 contributes to atrial remodeling and induces a pro-thrombotic state by increasing platelet production and coagulation factors, predisposing to AF
The activation of the IL-1β pathway, different from the NLRP3 inflammasome, could help to assess the appearance of NOAF in patients with ACS
Angiotensin II and reactive oxygen species (ROS) contribute to abnormal Ca^2+^ handling and the remodeling promotion of atrial tissue
In patients with AMI, elevated levels of galectin-3 are associated with a 4.4 times higher risk of developing atrial fibrillation
BNP independently predicts the occurrence of NOAF in STEMI patients treated by primary PCI
